# Iodine status five years after the mandatory salt iodization legislation indicates above requirement: a cross sectional study in Northwest Ethiopia

**DOI:** 10.1186/s40795-018-0261-8

**Published:** 2018-12-20

**Authors:** Molla Mesele Wassie, Zegeye Abebe, Amare Tariku, Ejigu Gebeye, Tadese Awoke, Azeb Atenafu Gete, Melkie Edris Yesuf, Yigzaw Kebede, Gashaw Andargie Biks, Shao Jia Zhou

**Affiliations:** 1 Department of Human Nutrition, Institute of Public Health, College of Medicine and Health Sciences, University of Gondar, Po Box: 196, Gondar, Ethiopia; 2 School of Agriculture Food and Wine, Faculty of sciences, The University of Adelaide, Adelaide, Australia; 3 Department of Epidemiology and Biostatistics, Institute of Public Health, College of Medicine and Health Sciences, University of Gondar, Gondar, Ethiopia; 4 Department of Health Service Management and Heath Economics, Institute of Public Health, College of Medicine and Health Sciences, University of Gondar, Gondar, Ethiopia

**Keywords:** Iodine status, UIC, Goiter, School-aged children, Ethiopia

## Abstract

**Background:**

Iodine deficiency is one of a major nutritional problem. The study aimed to assess the iodine status of populations in Dabat district using median urinary iodine concentration (UIC) in school-age children (6–12 years) and compared the results with goiter prevalence.

**Methods:**

The study used a cross-sectional study design. The study was conducted in Dabat district, northwest Ethiopia in May 2016. Spot urine samples were used for the determination of UIC. Manual examination of the thyroid gland was performed to assess presence of goiter. The MBI international Rapid Test Kits (RTK) were used to determine the level of salt iodine content. Children aged 6–12 years were recruited from schools using a multistage stratified sampling. One-way Analysis of Variance (ANOVA) was used to compare mean of log-transformed UIC values among key variables. Significant was determined at *P*-value less than 0.05.

**Results:**

A total of 358 school age children enrolled to the study. The mean age of children was 10.8((Standard Deviation (SD) = 1.45) years and 56.7% were males. The median UIC was 235 μg/l ((Inter Quartile Range (IQR): 161, 320]. Excessive iodine intake and inadequate iodine intake was observed on 29.1 and 8.7% of school aged children, respectfully. The coverage of iodized salt use in school-age children were 66.8%. The UIC was higher in urban children than rural counterparts and in those used adequately iodised salt than inadequately iodized salt (*P* < 0.05). Thirty-four percent of school-age children had a goiter. The prevalence of grade 1 and grade 2 goiter was 26.5 and 7.5%, respectively. There was a poor agreement between UIC and goiter (*k* = 0.1) in classifying iodine status of populations.

**Conclusions:**

The study population is classified as above requirements by median UIC in school-age children but severe iodine deficiency by goiter prevalence. Further research investigating the agreement between UIC and goiter prevalence in classifying iodine status of populations with various iodine status is warranted.

## Background

Iodine is an essential nutrient and its deficiency leads to iodine deficiency disorders (IDDs) [1]. Sever iodine deficiency during pregnancy causes complications like abortion, stillbirth, and malformation and impairs the brain growth and development of children [2, 3]. Iodine deficiency is one of a major nutritional problem and its control is a global public health triumph [4]. Universal salt iodization and fortification of iodine with foods like bread have been showed to have a significant impact on improving iodine status of vulnerable population groups worldwide [1, 5–8].

In Ethiopia, the use of iodized salt for human consumption was first launched in late 1980’s, but interrupted after Eritrea became an independent state [9, 10]. In February 2011 the government of Ethiopia passed a comprehensive legislation which mandated the use of iodized salt for human consumption. The WHO recommends that the coverage of iodized salt utilization at the household level should be above 90% to achieve elimination of iodine deficiency in countries with salt iodization program [11]. However, a nationwide survey in 2014 by Ethiopian Public Health Institute (EPHI) showed a national iodized salt coverage of 88.8% at the household level but the level of iodine in the table salt was adequate in only 53.9% of the households [12]. the 2016 Ethiopian Demographic and Health Survey reported 89.3% households used iodised salt [13]. The proportion of households using adequately iodized salt after introduction of the USI program were 29.7% in Lay Armachiho district [14], 33.2% in Dabat district [15], 28.9% in Gondar town [16], and 26.2% in Shebe Senbo district [17]. High prevalence of goiter [14, 17–21] and median UIC < 100 μg/l [17, 19, 21–23] was also reported across the country pre-and post-mandatory salt iodization in 2011. On the other hand, excessive iodine intake in the Ethiopian Somali regional state has also been reported [24, 25].

The introduction of mandatory iodized salt program did not improve the iodine status of populations in Northwest Ethiopia [14, 16, 18, 22]. A recent study by Abebe et al. in 2016, reported a goiter prevalence of 29.1% in school aged children which indicated iodine deficiency in Dabat District in Northwest Ethiopia [18]. UIC and goiter prevalence in school aged children are both recommended markers of populations’ iodine status [1, 26]. However, UIC is a marker of recent iodine intake, thus suitable to be used to detect recent changes in iodine status of populations while goiter is both a clinical and epidemiological marker that reflects longer term iodine deficiency [26, 27]. There is no any UIC data on school aged children in Dabat district.

Hence, the current study assessed the iodine status in Dabat District and assess agreement between two markers of iodine status of the population namely the median UIC and prevalence of goiter in school-age (6–12 years) children.

## Methods

### Study design and setting

A school-based cross-sectional study was conducted in May 2016, in Dabat District, northwest Ethiopia. The district is 821 km from Addis Ababa, the capital city of Ethiopia. It has 26 rural and four urban Kebeles (*smallest administrative unit in Ethiopia) with a* total population of 175,737*.* The altitude of the district ranges from 1000 to 2500 m above the sea level. Cereals, such as maize, sorghum, wheat, and barley are the main staple crops cultivated in the district. The study was conducted at Dabat Health and Demographic Surveillance System (HDSS) site which covers 13 randomly selected kebeles (4 urban and 9 rural) and stratified by ecological zones as highland, middle land and lowland [18, 28, 29].

### Study participants

All school children aged 6–12 years who lived in the HDSS site and attending school during the study period were eligible for the study. The sample size of the study was calculated using Open Epi version 2.3 with the following assumptions: 29.1% prevalence of goiter in school aged children in Dabat district [18], 95% Confidence Level, a 10% non-response rate and a 5% margin of error. The minimum sample size of 349 was estimated.

### Participants’ recruitment

A multistage cluster sampling technique was employed to select participants in order to recruit a representative population sample. Initially, schools within the HDSS site were stratified into urban and rural based on their geographic location. Then, five rural and one urban schools from each HDSS site were randomly selected to be included in the survey by lottery method. List of all students aged 6–12 years and enrolled in the schools were identified from the schools’ registry. All Students aged 6–12 in the included schools were randomly selected to take part in the survey using a systematic random sampling technique. The total number of students selected from each school was proportionate-to-student size of the schools.

### Data collection

Physical examination of goiter and collection of urine samples from children aged 6–12 years were conducted at schools. Information on child’s name and home address were recorded and used to reach the parents of children. Questionnaire was used to collect the socioeconomic and nutrition related information in children and their parents. The pilot testing of the Questionnaire was done on 22 school aged children and their parents in the district who were not part of the study. Some wordings of the Questionnaire were adapted to the local context and total amount of time required for interview and child assessments defined following the pilot test.

### Assesement of urinary iodine concentration

A 10 ml spot urine sample was collected from each child using disposable plastic cup and immediately transferred to cap polyethylene test tube and labeled with an identification number. The urine samples were stored in cold chain until the end of the day and then it were stored at below -20^o^c refrigerator until transported to Ethiopian Public Health Institute, a national accredited laboratory, for analysis. The UIC was analyzed using the Sandell Kolthoff reaction [26] method recommended by WHO/ICCIDD/UNICEF. Iodine status was classified according to WHO in which iodine deficiency was defined as UIC < 100 μg/l, adequacy at 100–199 μg/l, above requirement at 200–299 μg/l and iodine excess at > 300 μg/l [26].

### Assessment of goiter

Goiter was assessed by a qualified health officer by inspection and palpation. Training on the techniques used to perform thyroid examination and the WHO grading of goiter was given for the assessors before the actual data collection. The physical examination was performed following techniques recommended by the WHO [26]. Goiter was defined as; grade 0 if it is not palpable or visible, grade 1 if it is palpable but not visible and grade 2 if it is visible and palpable [26].

### Assessment of household salt iodine content

A tablespoon of salt was collected from each household and was kept in a dark, dry and cool place to inhibit iodine evaporation. The MBI international Rapid Test Kit (RTK) obtained from UNICEF Ethiopia were used to measure iodine content of the salt semi-quantitatively [26]. Using this method, level of iodine in the salt was classified as 0 ppm (PPM), < 15 PPM and ≥ 15 PPM. A level of iodine ≥15 ppm was categorized as adequate iodine utilization [26]. The coverage of iodized salt use was calculated as the proportion of households having salt iodine content > 0 ppm among all households included in the study.

### Assessment of socio-economic status (SES)

A structured interviewer-administered questionnaire was used to collect data on SES of parents 1 week before assessment of goiter and collection of urine sample. Data were collected by health extension workers at households by asking about the sociodemographic characteristics, wealth index, behavioral characteristics and dietary intake/pattern of parents and the child. Questions assessing the household wealth index and child’s diet diversity score were taken from the Ethiopian Demographic and Health Survey (EDHS) questionnaire [18, 30]. Principal Component Analysis (PCA) was used to assess the wealth index from household assets. Variables related to the size of agricultural land, the amount of grains harvested, house types and the number of livestock were asked to calculate the wealth index. Then, a code between 0 and 1 given to the variables and analyzed using the PCA. The factors were ranked in to the lower, medium and higher tertiles. Finally, these tertiles were categorised as rich, medium and poor. Food frequency questionnaire was used to collect the food intake of school children in the preceding day of the survey.

### Dietary assessment

Intake of foods from the seven food groups which include starchy staples, organ meat, flesh meat and fish, egg, green leafy vegetables, vitamin A rich fruits and vegetables, other fruits and vegetables were recorded to the dietary recall questionnaire by asking the Mothers about the types of foods eaten by the child in the preceding full day of the survey. Dietary diversity score (DDS) was calculated from the one-day (24 h) dietary intake data. The dietary recall questionnaire was validated and has been applied to determine dietary diversity score in Ethiopia [30]. The minimum DDS which is optimal for child health and development is four, hence children scoring bellow four were categorized as having poor DDS [13, 31]. The dietary history on major food sources of iodine including milk, meat, egg and some goitrogenic foods including cabbage and millet were recorded. The source of drinking water for the households was also collected to observe the degree of iodine deficiency across the source of drinking water. Nine yes/no questions on mothers’ knowledge regarding iodine deficiency disorders, benefits of iodine intake, food sources of iodine and proper utilization of iodized salt has been asked [14, 18]. The questions were coded between 0 and 1 and analyzed using PCA to produce factor scores. The factors summed up and ranked to a higher and lower tertiles. Finally, Mothers’ knowledge was categorized as poor or good.

### Data management and statistical analysis

Data were checked and entered into EPI-Info version 7 and exported to SPSS version 20 statistical software for further analysis. The UIC data were not normally distributed and were log-transformed for analysis. The kappa coefficient (k) was calculated to assess the agreement in defining iodine status between goiter and UIC. The strength of agreement was categorized as poor for *k* value 0.0–0.20, fair for *k* value 0.21–0.40, moderate for k value 0.41–0.60, good for *k* value 0.61–0.80, and excellent for *K* value 0.81–1.00 [32]. One-way Analysis of Variance (ANOVA) was used to compare mean of log-transformed UIC values among key variables which include place of residence, maternal educational status, wealth index, salt iodine content, presence of goiter, and child’s diet score. Significant was set at a *P*-value less than 0.05.

## Results

### Characteristics of participating families

A total of 358 children agreed to take part in the study. The mean age of children was 10.8 years (Standard Deviation, SD: 1.5) and 56.7% were males. Majority of children were from rural schools and were Orthodox Christians. 87.4% of mothers were married and 78.5% were unable to read and write. The main source of drinking water was from springs and protected wells. Half of the mothers had good knowledge on utilization of iodized salt. Only 35 (9.8%) children had a good dietary diversity score (Table 1).

**Table 1:** Sociodemographic and economic characteristics of study sample by median urinary iodine concentration (*N* = 358)

Variable	N (percentage)	Median UIC in μg/l (IQR)
Age in years		
< 8	36(10.1)	237.5[150.3235.4]
8–10	102(28.5)	206.8[155.6303.1]
> 10	220(61.5)	239.7[161.7327.6]
Sex		
Male	155 (43.3)	238.1 [156.1, 326.9]
Female	203 (56.7)	228.0 [161.8, 314.8]
Residence		
Urban	69 (19.3)	254.8 [207.8, 319.1]^*^
Rural	289 (80.7)	228.0 [151.1, 321.1]^*^
Religion		
Orthodox	351 (98.0)	235.4 [161.3, 323.5]
Muslim	7 (2.0)	230.4 [120.2, 260.6]
Maternal educational status		
Illiterate	284(79.3)	235.8[161.3, 314.6]
Elementary school(grade 1–8)	40 (11.2)	226.8 [165.9352.8]
High school(grade 9–10)	19 (5.3)	236.2 [139.7339.4]
Preparatory school(grade11–12), College/ university	15(4.2)	234.2[152.6, 279.2]
Paternal educational status		
Illiterate	239(66.7)	236.5 [161.7, 320.1]
Elementary school(grade1–8)	79 (22.1)	220.5 [147.7, 313.0]
High school(grade 9–10)	16 (4.5)	254.4 [231.4, 343.6]
Preparatory school(grade11–12), College/ university	24(6.7)	227.5[144.1, 353.1]
Preparatory school(grade11–12)	8 (2.2)	249.3 [159.5, 406.0]
College/ university	16 (4.5)	209.4 [139.8, 333.5]
Maternal marital status		
Single ^a^	9 (2.5)	236.5 [175.6, 312.4]
Married	313 (87.4)	234.5 [159.6, 324.0]
Divorced	15 (4.2)	256.7 [165.9, 324.8]
Widowed	21 (5.9)	211.0 [124.2, 284.9]
Wealth Index		
Poor	118 (33.0)	231.7 [163.3304.6]
Medium	121 (33.8)	224.1 [140.4322.7]
Rich	119 (33.2)	238.6 [169.1330.5]

*IQR* Inter quartile range, ^a^Never Married ^*^P < 0.001 from the ANOVA test

### Iodine status of participants

The median UIC of study subjects was 235.0 μg/l(Inter Quartile range, IQR: 161.3, 320.2). As depicted in Table 2 the prevalence of insufficient iodine intake as indicated by UIC less than 100 μg/l was 8.7% (95% CI: 6.2, 12.1) and 29.1% of school-age children had excessive iodine intake. The overall prevalence of goiter in school aged children was 34%. The prevalence of grade 1 and grade 2 goiter was 26.5 and 7.5%, respectively. There were no difference in median UIC between maternal knowledge levels, DDS and goiter grades. The median UIC was higher in males, aged > 10 years, and those lived in urbans (Table 1).

**Table 2:** Classification of iodine status of school-age children based on WHO criteria, Dabat District, 2016

Median UIC(μg/l)	Number (%)	Iodine status
< 20	3 (0.8)	Severe deficiency
20–49	4 (1.1)	Moderate deficiency
50–99	24 (6.7)	Mild deficiency
100–199	100 (27.9)	Adequate
200–299	123 (34.4)	Above requirements
> 300	104 (29.1)	Excessive

*UIC* Median urinary iodine concentration

### Coverage and adequacy of iodized salt use

The coverage and adequate level of iodized salt use in school-age children were 66.8 and 30.7%, respectively. The median UIC was higher in children from households with level of salt iodine content ≥15 ppm (243.6 μg/l, IQR: 195.4, 347.1) as compared to those < 15 ppm (229.5 μg/l, IQR: 145.8, 313.6) and 0 ppm (95.0 μg/l, IQR: 67.1, 248.5) (Table 3).

### Agreement between urinary iodine concentration and goiter prevalence

As shown in Table 4 the agreement between goiter and median UIC was assessed using kappa coefficient. There was a poor agreement between median UIC and goiter prevalence (*k* = 0.1, *P* value = 0.01) to classify iodine status in school aged children.

**Table 3:** Median urinary iodine concentration of study sample by level of iodine in salt, dietary diversity score, goiter grade and source of drinking water (*N* = 358)

Variables	N (percentage)	Median UIC in μg/l (IQR)
Salt iodine level(ppm)		
Not iodized (0)	9 (2.5)	95.0 [67.1, 248.5]
Inadequately iodized (< 15)	239 (66.8)	229.5 [145.8, 313.6]^*^
Adequately iodized (≥15)	110 (30.7)	243.6 [195.4, 347.1]^*^
Maternal knowledge of iodized salt use		
Poor	180 (50.3)	230.0 [154.2, 309.6]
Good	178 (49.7)	237.1 [161.8, 329.5]
Goiter grade		
No goiter	236 (65.9)	229.7 [161.9304.3]
Grade 1	95 (26.5)	253.4 [145.8344.9]
Grade 2	27 (7.5)	215.6 [161.3321.9]
Source of drinking water		
Tap(in the house/compound)	40 (11.2)	241.2 [163.0,346.5]
Public tap	31 (8.7)	242.3 [152.9, 320.2]
Protected well	71 (19.8)	220.8 [139.8, 334.8]
Unprotected well	21 (5.9)	208.2 [163.4, 297.3]
Protected spring	122 (34.1)	246.8 [169.8, 340.8]
Unprotected spring	64 (17.9)	227.6 [137.5, 296.0]
River	9 (2.5)	243.9 [215.2, 259.3]
Milk intake		
Once and above per week	50 (14.0)	242.1 [141.2351.6]
No	308 (86.0)	234.1 [161.8313.5]
Meat intake		
Once and above per week	60 (16.8)	252.8 [145.8, 346.0]
No	298 (83.2)	230.3 [161.6, 312.9]
Egg intake		
Once and above per week	27 (7.5)	250.9 [154.8, 351.6]
No	331 (92.5)	230.4 [161.3, 314.8]
Cabbage intake		
Once and above per week	22 (6.1)	227.0 [118.6, 385.1]
No	336 (93.9)	235.0 [161.7, 320.1]
Millet consumption		
Once and above per week	7 (0.2)	131.9 [93.2, 320.1]
No	351 (98.0)	235.4 [161.7, 321.9]
DDS		
< 4 scores	323 (90.3)	234.5[161.6314.0]
≥4 scores	35 (9.8)	236.2 [139.8, 351.6]

*DDS* Dietary diversity score, *IQR* Inter quartile range, *ppm* Parts per million, *P < 0.001 from the ANOVA test

**Table 4:** Agreement between UIC and goiter to diagnose iodine deficiency in school age children

	With goiter	No goiter	Total	Kappa coefficient^a^	*P* value
UIC < 100 μg/l	17	14	31	0.098	0.011
UIC ≥100 μg/l	105	222	327		
Total	122	236	358		

^a^Kappa is calculated for iodine deficiency in 2 X 2 table (UIC < 100 μg/l vs. presence of goiter) and numbers in cells are reported

## Discussion

Our study is the first to assess iodine status using two recommended population markers of iodine status in northwest Ethiopia. We found that the population was classified as above requirements based on the median UIC but severe iodine deficient based on the goiter prevalence.

The median UIC in school aged children in the current study was above requirements and higher than studies done post-salt iodization program in Ethiopia [17, 19, 22, 23]. The reason why the median UIC was higher than other areas may be due to variation in dietary sources of iodine and the coverage of iodised salt use in study populations. However, the intake of the main food sources of iodine is low in Ethiopia [13] in general and the study area [14] in particular. The variation in the dietary sources of iodine is unlikely to cause such substantial variations in the median UIC across the studies. Therefore, the difference in the coverage and utilization of iodized salt could be the cause of disparity in the median UIC across different regions in the country. The geographical location may affect access to iodised salt in populations. The current study includes both urban and rural areas in terms of location, and both highlands and lowlands in altitude, however, studies in other areas were conducted mainly in rural and populations living in highlands.

Based on the current study the salt iodization program was effective in combating iodine deficiency in the study population. Universal salt iodization also improves iodine status of other countries [1, 11, 26]. Iodine intake was optimal in the majority of participants included in the study, one-third of them were found with excessive iodine intake above 300 μg/l. Excessive iodine intake during early childhood may potentially lead to iodine-induced thyroid dysfunction; both hypothyroidism and hyperthyroidism [33, 34]. A higher concentration of iodine from a poorly controlled salt iodisation program may be the source of excess iodine in these population. Besides, other sources of dietary iodine including water need to be explored in the food-chain of the study area to monitor intake and ensure that the population is not adversely exposed to excessive levels of iodine.

The prevalence of goiter is comparable with similar studies [14, 18] in the study area which reported iodine deficiency post-iodine fortification. However, there is a discrepancy in reporting iodine status in the same population at the same point in time among these two epidemiological markers of iodine status: prevalence of goiter and median UIC. Discrepancy in classifying iodine status in populations between goiter prevalence and median UIC also reported elsewhere [27, 35]. Median UIC indicates recent intake and it is a sensitive marker to detect recent changes in iodine status in the population. Conversely, the prevalence of goiter indicates medium to long term iodine intake and may not be sensitive to recent changes in the iodine status of populations [11, 26, 27]. The current study was conducted 5 years post mandatory iodine fortification in Ethiopia and presumably the two markers need to agree on assessing the degree of iodine status in the population. However, the severe iodine deficiency by goiter and above requirements by median UIC shows the iodised salt was introduced only recently. Intake of goitrogenic foods other than iodine deficiency may lead to goiter in populations [36, 37]. Moreover, assessment of goiter by palpation is subjective and prone to bias. However, the assessors were qualified, and training was given before commencement of data collection. It appears that total goiter rate may not be an appropriate epidemiological tool to monitor iodine status of the population shortly after universal salt iodization program.

The WHO, UNICEF, and ICCIDD jointly recommend the level of iodine should be 15–40 ppm at a household level and 20–40 ppm in the retails to fulfil iodine requirement of the population. Moreover, to demonstrated successful use of iodised salt in the study population, above 90% of households, should use adequately iodised salt which is above 15 ppm and bellow 40 ppm [26]. Even though the population in the present study were above requirement based on the median UIC, salt iodine level was adequate only in 30.7% of households. In this study, 29.1% of school-age children had excessive iodine intake > 300 μg/l and the median UIC was significantly higher in children from households with the level of salt iodine content was ≥15 ppm. This shows salts with iodine content ≥15 ppm may have extreme high concentrations of iodine that needs a quantitative measurement to confirm the exact iodine concentration in table salts. However, we have used a rapid test kits to measure iodine concentration in the salts and classify adequacy of iodine qualitatively. This warrants assessment of salt iodine contents using a quantitative marker such as a titration method. This finding is consistent with other study in the same population which reported 33.2% households used adequately iodised salt post fortification [15]. Higher proportion of inadequate salt iodine content among households in the study area indicates a possibility of fluctuations in iodised salt supply in the community or availability of non-iodised salt in the local market or poor utilization of iodised salt including use of open containers to store salt, exposing the salt to sunlight and storage of salt for long period of time at the household level. This warrants further research measuring salt iodine level at retail shops and salt manufacturing sites. Even though we did not assess the iodine intake using iodine specific food frequency questionnaire, the intake of main sources of foods including fish, eggs and dairy products are low in the population and a close monitoring of the utilization of iodized salt in the study area is warranted.

The study is a cross-sectional survey and might not show the trend overtime in iodine status since the introduction of iodine fortification program in the study population. The study employed a multistage cluster sampling procedure but did not adjust the sample size using the design effect to minimize the clustering effect in the inferential statistics. However, the calculated sample size is adequate to measure median urinary iodine concentration in the study population. There may be intra-assessor variation on measurement of goiter in school aged children. We used a semi-quantitative method to assess the level of iodine in the salt and may not exactly quantify the concentration of iodine in the salt.

## Conclusion

The iodine status of the study population is classified as above requirements by median UIC in school-age children but severe iodine deficiency by goiter prevalence. Regular monitoring of the level of household iodised salt and median UIC in school-age children and other vulnerable groups like pregnant and breastfeeding women is recommended. Continuous monitoring of iodine status of the population helps to detect potential harms of both iodine deficiency and excess. Studies investigating causes of goiter other-than iodine deficiency and the iodine content of staple foods and drinking water in the study area are also warranted. Further research investigating the agreement between median UIC and goiter prevalence in classifying iodine status of populations with various iodine status is warranted.

## Abbreviations

ICCIDD: International Council for Control of Iodine Deficiency Disorder
IDD: Iodine Deficiency Disorder
UIC: Urinary Iodine Concentration
UNICEF: United Nation Children’s Fund
WHO: World Health Organization
